# Neural Reward Processing in Digital Addiction: A Dynamical Systems Theory of Reward Instability

**DOI:** 10.3390/brainsci16060584

**Published:** 2026-05-29

**Authors:** Anna Makarewicz, Remigiusz Recław, Elżbieta Grzywacz, Krzysztof Chmielowiec, Łukasz Jaworski, Marta Kuczak-Wójtowicz, Jolanta Chmielowiec

**Affiliations:** 1 Department of Hygiene and Epidemiology, Collegium Medicum, University of Zielona Góra, 28 Zyty St., 65-046 Zielona Góra, Poland; makarewicz81@gmail.com (A.M.); chmiele@vp.pl (K.C.); 2 Independent Laboratory of Behavioral Genetics and Epigenetics, Pomeranian Medical University in Szczecin, Powstancow Wielkopolskich 72 St., 70-111 Szczecin, Poland; 3 Department of Medical Sciences and Public Health, Gdansk University of Physical Education and Sport, Kazimierza Górskiego 1 St., 80-336 Gdansk, Poland; 4 Independent Public Specialist Health Care Facility “ZDROJE”, Mączna 4 St., 70-780 Szczecin, Poland; elagrzywacz@me.com (E.G.); martakuczak@wp.pl (M.K.-W.); 5 Cardiac Surgery Department, Specialized Hospital in Wejherowo, Jagalskiego 10 St., 84-200 Wejherowo, Poland; ljaworski75@gmail.com; 6 Department of Nursing, Collegium Medicum, University of Zielona Góra, 28 Zyty St., 65-046 Zielona Góra, Poland; j.chmielowiec@inz.uz.zgora.pl

**Keywords:** behavioral addiction, reward processing, reward instability, reward landscape, attractor dynamics, reinforcement learning, dopamine signaling, computational psychiatry, digital addiction, digital phenotyping

## Abstract

**Highlights:**

**What are the main findings?**

- This review introduces Reward Instability Theory, proposing that digital addiction may emerge as an attractor-like state within distorted reward landscapes shaped by high-density and high-variance reinforcement signals.
- The article outlines the Behavioral Reward Instability Index (BRII) as a heuristic systems construct integrating individual reward sensitivity, environmental reinforcement structure, and behavioral variability.

**What are the implications of the main findings?**

- The framework shifts addiction research from static symptom descriptions toward dynamic models of behavior involving non-linear transitions, persistence, and reduced behavioral flexibility.
- If empirically validated, BRII-informed approaches may support digital phenotyping research and help identify periods of increased vulnerability for adaptive intervention timing.

**Abstract:**

Behavioral addiction in digital environments is an increasingly relevant neurobehavioral phenomenon characterized by persistent engagement with high-frequency, algorithmically optimized reward stimuli. Although neural correlates of addictive behaviors have been widely studied, current models only partly explain how modern reinforcement environments reorganize behavior at the systems level. This review introduces Reward Instability Theory, a conceptual dynamical systems framework proposing that behavioral addiction may emerge as an attractor-like state within distorted reward landscapes shaped by high-density and high-variance reinforcement signals. The model shifts focus from static behavioral descriptions toward a systems account of motivation involving reinforcement learning, salience attribution, executive control, and environmental reward structure. We propose that digital environments may increase reinforcement density and reward variance, promoting dominant reward peaks and reducing behavioral diversity. To formalize these dynamics, we outline the Behavioral Reward Instability Index (BRII) as a heuristic systems construct integrating individual reward sensitivity, environmental reinforcement structure, and behavioral variability. The framework also situates established addiction models—including incentive sensitization, habit formation, and allostatic regulation—within a shared dynamical architecture. In addition, digital phenotyping is discussed as a potential empirical strategy for testing reward instability, while acknowledging limitations related to signal noise, ecological validity, bias, and privacy. This model is intended to explain problematic patterns characterized by reduced behavioral flexibility, persistence despite negative consequences, and functional impairment, rather than all forms of frequent digital use. Attractor-like terminology is used throughout as a conceptual heuristic to describe behavioral persistence and reduced flexibility, rather than as evidence of formally verified mathematical attractors.

## 1. Introduction

Digital environments have created an emerging and highly influential context of human–reward interaction. Social media platforms, online gaming systems, and algorithmically curated content streams deliver reinforcement signals at exceptional levels of temporal density, variability, and scale. Rather than simply increasing exposure to rewarding stimuli, these systems may alter the statistical structure of reward distributions encountered by the brain [1,2,3].

Problematic engagement with digital platforms has become an increasingly relevant mental health concern, particularly among adolescents and young adults. Excessive or dysregulated digital engagement has been associated with emotional distress, sleep disruption, attentional difficulties, functional impairment, and compulsive behavioral patterns [3,4].

Conceptual clarification: Throughout this manuscript, we distinguish between normative high engagement—characterized by frequent but volitional, flexible, and contextually adaptive digital use—and problematic engagement, defined by persistent behavioral patterns that (i) substantially reduce behavioral flexibility, (ii) persist despite awareness of negative consequences, and (iii) produce clinically significant distress or functional impairment [3,4,5]. The term behavioral addiction is used to describe cases where problematic engagement exhibits features consistent with addictive processes (e.g., loss of control, craving, withdrawal-like distress), while recognizing that digital addiction is not formally recognized as a distinct diagnostic category in current psychiatric classification systems [3,4,5]. Importantly, the present framework is intended to explain the emergence and persistence of problematic patterns—not to pathologize all forms of frequent digital use, which may serve adaptive social, educational, recreational, or occupational functions [3,4].

Converging evidence suggests that such environments strongly engage core neurobiological systems involved in motivation and reward processing. High-frequency and variable reinforcement signals may repeatedly recruit dopaminergic learning mechanisms, enhance salience attribution processes, and challenge executive control systems responsible for behavioral regulation [4,5,6,7]. These interactions have been associated with heightened reward responsivity alongside reduced regulatory control in individuals exhibiting problematic digital engagement, supporting the view that some forms of excessive digital behavior may share features with behavioral addiction [8,9].

However, existing theoretical frameworks have largely focused on specific mechanisms, including dopaminergic sensitization, impaired executive control, and maladaptive habit formation. Although these models provide important insights into neural processes underlying addictive behavior, they only partly explain how environmental reward structures may reorganize behavior at the level of system dynamics [10,11,12,13].

To illustrate these limitations, consider how traditional addiction models interpret problematic digital engagement. Trait-based frameworks typically attribute high-frequency platform use and distress during abstinence to stable individual vulnerabilities such as impulsivity or dopaminergic reward sensitivity [3,4,5]. However, such interpretations struggle to explain why the same individuals show markedly different engagement patterns across platforms, why symptoms fluctuate dramatically following environmental changes (algorithm updates, notification settings), or why brief behavioral interventions produce rapid shifts that trait models would not predict. Cross-sectional symptom-count approaches may not fully capture these dynamic, context-dependent patterns [3,4]. This gap reflects a broader conceptual limitation: while existing models effectively describe individual components of addictive behavior, they provide limited insight into the emergent, system-level dynamics arising from the interaction between neurobiological mechanisms and engineered reward environments [11,13]. In particular, current accounts do not fully explain why sustained exposure to high-density digital reinforcement may progressively narrow behavioral repertoires, or why such patterns become increasingly stable and resistant to change over time [14,15,16].

To address this gap, we adopt a conceptual dynamical systems perspective in which behavior is modeled heuristically as trajectories evolving within a structured reward landscape. Within this framework, behavioral selection emerges from probabilistic competition among actions, shaped by reinforcement learning processes and constrained by both neurobiological parameters and environmental inputs [17,18,19]. Dynamical terminology (e.g., attractor states, phase transitions) is used as a theoretical organizing framework rather than as empirically validated descriptions of neural or behavioral processes.

In this review, we propose that behavioral addiction in digital environments may be conceptualized as an attractor-like state emerging within distorted reward landscapes shaped by high-density reinforcement signals [16,19,20]. At the same time, high engagement with digital environments should not be equated with pathology, as adaptive, recreational, and socially meaningful forms of use may arise within the same platforms. The framework is specifically focused on understanding persistent, rigid, and dysregulated behavioral patterns characterized by reduced disengagement capacity and continued engagement despite negative consequences—dynamics that differ qualitatively from normative high-frequency use that remains flexible and responsive to changing contexts [3,4,5,20,21]. This perspective reframes addiction not as a discrete pathological condition, but as a system-level phenomenon influenced by reward landscape dynamics rather than isolated behavioral or neural dysfunctions [11,14,15,16]. The mechanisms underlying this distortion are detailed in Section 3.

Importantly, this framework is not intended to replace existing models of addiction. Rather, it offers an integrative architecture that links established mechanisms—such as incentive sensitization, habit formation, and allostatic adaptation—within a unified dynamical perspective [20,21,22]. To formalize these processes, we introduce the Behavioral Reward Instability Index (BRII) as a conceptual construct capturing interactions between biological reward sensitivity, environmental reinforcement structure, and behavioral variability. Unlike linear behavioral metrics, the BRII is framed as a non-linear system variable reflecting relative proximity to instability and attractor-like dominance [14,15,16].

Finally, although the present work is primarily theoretical, we discuss digital phenotyping as a potential empirical approach for examining reward-driven behavioral dynamics in real-world settings, while acknowledging important limitations related to signal noise, ecological validity, and data bias [23,24].

## 2. Neural Mechanisms of Reward Processing in Digital Addiction

Understanding behavioral addiction in digital environments requires moving beyond isolated neural mechanisms toward a systems-level account of interacting neurocomputational processes. Human motivation arises from the dynamic interplay between reinforcement learning systems, salience attribution networks, and executive control mechanisms, all operating in continuous interaction with environmental inputs [25,26,27].

Importantly, these processes are further shaped by individual differences in reward sensitivity, including genetic, neurobiological, and trait-related factors that may influence how strongly reinforcement signals affect behavior [21,22]. Such variability helps define the effective parameter space within which behavioral dynamics unfold, contributing to differences in susceptibility to maladaptive reward-driven patterns.

**Figure 1:**
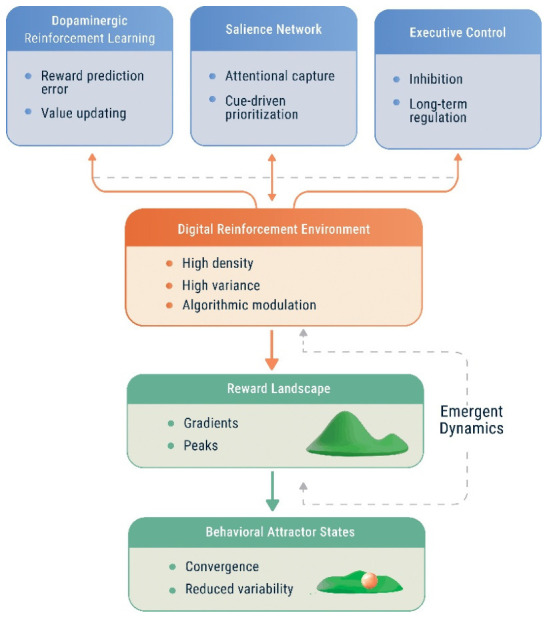
Neurocomputational architecture of reward-driven behavior in digital environments. The diagram illustrates the systems-level interaction between neurobiological processes and environmental reinforcement structure. The Digital Reinforcement Environment (orange box, **bottom**) serves as the primary input, delivering high-density and high-variance reinforcement signals characteristic of algorithmically optimized platforms. Upward arrows indicate that these environmental signals simultaneously engage three core neurobiological systems (blue boxes, **top**): dopaminergic reinforcement learning (reward prediction error and value updating), the salience network (attentional capture and cue-driven prioritization), and executive control (inhibition and long-term regulation). Solid arrows indicate directional information flow between system components, whereas dashed arrows represent feedback dynamics and emergent reorganization of the reward landscape. Together, these interactions modulate the topology of the reward landscape (green box) by shaping reward gradients and peaks, which in turn influence behavioral dynamics. Under sustained high-density reinforcement, the system may become progressively biased toward dominant reward states, increasing the likelihood of behavioral convergence toward attractor-like configurations (**bottom** green box). Emergent dynamics (feedback loop, **right**) reflect the non-linear interaction between environmental inputs and system-level reorganization. This framework emphasizes that behavioral addiction may arise not from isolated neural dysfunction, but from the coupled interaction between reward-sensitive neurobiological processes and engineered digital environments.

The interaction between core neurobiological systems and structured environmental reinforcement is illustrated schematically in Figure 1. In this model, dopaminergic reinforcement learning, salience attribution, and executive control processes interact with high-density and high-variance digital reinforcement signals. Together, these influences may shape the topology of the reward landscape by modulating reward gradients, attentional weighting, and behavioral flexibility. Under sustained reinforcement, the system may become progressively biased, increasing the likelihood that behavioral trajectories converge toward dominant attractor-like states.

Digital environments may exert a disproportionate influence on behavior because they can simultaneously and repeatedly engage all three systems under conditions of rapid, stochastic, and persistent reinforcement [4,5,6,7,23,24]. This creates a regime in which learning processes are continuously updated, attentional systems are repeatedly captured, and regulatory control mechanisms are persistently challenged. From a dynamical perspective, such interactions may promote sustained perturbation of system equilibrium and progressively bias behavior toward highly reinforced reward states.

### 2.1. Dopaminergic Reinforcement Learning Under High-Density Stimulation

At the core of motivational regulation lies the dopaminergic reinforcement learning system, in which midbrain neurons encode reward prediction errors that update expected value representations of actions, cues, and stimuli [1,6]. These processes are central to reward learning and adaptive decision-making, particularly within mesolimbic pathways linking the ventral tegmental area and ventral striatum [1,6].

Digital environments may substantially modify this reward-learning regime. Variable reinforcement schedules—implemented through notifications, social feedback, intermittent rewards, and algorithmically curated content—can generate persistent and stochastic prediction-error signals that prolong dopaminergic responsivity over extended periods [17,18].

As a consequence, the system may operate under conditions of continuous micro-reinforcement, promoting amplification of reward representations associated with frequently reinforced behaviors, particularly within the ventral striatum and related corticostriatal circuits [3,21]. Repeated exposure to such contingencies may also bias valuation processes toward immediately accessible rewards [21,22].

Within the reward landscape framework, these processes may be conceptualized as a steepening of reward gradients, thereby increasing the probability that behavioral trajectories converge toward highly rewarded regions of the landscape.

### 2.2. Salience Attribution and Attentional Capture

Reinforcement learning operates in conjunction with neural systems responsible for assigning attentional priority to stimuli. The salience network—centered on the anterior insula and anterior cingulate cortex—plays a critical role in detecting behaviorally relevant signals, monitoring internal and external demands, and allocating cognitive resources accordingly [5,7].

Digital environments are often designed to strongly engage this system [13]. Notifications, visual cues, novelty signals, and social validation cues may function as salience amplifiers and predictive stimuli, thereby enhancing attentional capture and reinforcing reward-learning processes [13].

Repeated exposure to such stimuli may recalibrate salience attribution processes, contributing to persistent attentional biases toward digital cues and increasing susceptibility to context-dependent triggers. As a result, users may become more likely to re-engage with previously reinforced behaviors even in the absence of strong intrinsic motivation [4,7,13].

Within the reward landscape framework, these processes may be conceptualized as a reweighting of perceptual and motivational inputs, increasing the prominence, accessibility, and behavioral pull of specific reward peaks.

### 2.3. Executive Control and System Imbalance

Adaptive behavior depends on executive control systems capable of regulating impulsive responses, maintaining goals, and supporting long-term decision-making. These systems, primarily associated with prefrontal cortical regions—including the dorsolateral prefrontal cortex, orbitofrontal cortex, and anterior cingulate cortex—provide top-down modulation of reward-driven processes [5,10].

In addiction-related conditions, a characteristic imbalance may emerge between bottom-up reward mechanisms and top-down regulatory control. Empirical studies suggest reduced functional connectivity and less efficient coordination between prefrontal control regions and reward-processing systems in individuals with problematic digital engagement [14,15].

Such dysregulation may impair inhibitory control, increase cue-reactivity, and reduce the capacity to prioritize delayed or non-digital rewards over immediately available reinforcement. From a behavioral perspective, this may contribute to repetitive engagement despite awareness of negative consequences [5,10].

Within the present framework, this imbalance can be interpreted as a shift in the relative weighting of forces within the reward landscape, whereby dominant reward peaks progressively reduce the effective accessibility of alternative behavioral trajectories.

### 2.4. Individual Differences in Reward System Dynamics

Beyond circuit-level mechanisms, individual differences in susceptibility to reward-driven behavior are also influenced by genetic, neurobiological, and trait-related variability affecting dopaminergic signaling, prefrontal regulation, impulsivity, and reward sensitivity [21,22]. Such factors may help explain why similar digital environments produce markedly different behavioral outcomes across individuals.

Rather than determining behavior directly, these influences are better understood as modulators of core system parameters, including reward sensitivity, responsiveness to reinforcement variability, delay discounting tendencies, and the efficiency of executive control. This perspective aligns with contemporary approaches in computational psychiatry, in which biological variability is conceptualized as influencing system parameters rather than exerting deterministic effects [25,26,27].

The impact of such variability may become particularly relevant in modern environments characterized by high-density and high-variance reinforcement. Under these conditions, even subtle differences in dopaminergic responsivity, inhibitory control, or sensitivity to immediate reward may contribute to disproportionate divergence in behavioral trajectories over time [25,26,27].

Accordingly, vulnerability to maladaptive digital engagement may reflect the interaction between environmental reinforcement structures and pre-existing differences in reward-system dynamics, rather than any single biological determinant. Key factors potentially influencing reward sensitivity and system organization are summarized in Table 1.

Within the present framework, genetic and neurobiological influences are conceptualized as modulators of system-level parameters governing reward sensitivity, reinforcement learning gain, and executive regulation. Rather than directly determining behavior, they may shape the topology of the reward landscape and alter the probability and trajectory of convergence toward attractor-like states.

Beyond neurobiological factors, psychosocial and contextual influences may also substantially modulate reward landscape dynamics. Loneliness, social isolation, and unmet affiliation needs may increase the relative salience of digital social rewards, thereby steepening reward gradients associated with platforms offering social validation or parasocial connection [3,4,5,20,21]. Peer dynamics and social identity processes may shape which digital behaviors become normative and reinforced within specific social contexts [28,29]. Developmental stage—particularly adolescence, characterized by heightened reward sensitivity and peer influence—may create periods of elevated vulnerability to high-density reinforcement environments. Economic incentives (e.g., platform monetization strategies, influencer economies) and cultural variation in digital norms, social expectations, and regulatory frameworks may further influence both reinforcement structure and individual susceptibility. Importantly, these factors are not external to the reward landscape framework; rather, they may be understood as modulators of landscape topology, altering the relative prominence of specific reward peaks, the accessibility of alternative behavioral trajectories, and the baseline reward sensitivity of the system [14,15,16,30]. Future refinements of the framework should more explicitly formalize how psychosocial contexts interact with neurobiological parameters to shape system-level dynamics.

### 2.5. Integration with Core Theories of Addiction

Reward Instability Theory is intended as an integrative framework that situates established models of addiction within a shared dynamical structure rather than replacing them. From this perspective, influential theories may be viewed as describing complementary processes operating at different levels of reward-system organization.

Incentive sensitization theory emphasizes the progressive amplification of motivational salience associated with specific stimuli and cues [4]. Within the present framework, this process may be interpreted as a local steepening of reward gradients, increasing the behavioral pull of selected reward peaks.

Habit formation models describe the transition from goal-directed action to increasingly automatic and repetitive behavior through repeated reinforcement [8]. In dynamical terms, this may correspond to the stabilization and deepening of attractor-like states, making previously reinforced trajectories more likely to recur.

Allostatic models emphasize long-term shifts in baseline reward processing, often accompanied by reduced sensitivity to alternative rewards and compensatory behavioral seeking [10]. Within the reward landscape framework, such changes may be conceptualized as a broader deformation of the landscape that further biases behavior toward dominant reward regions.

Dual-process perspectives, which distinguish impulsive reward-driven responding from reflective regulatory control, may also be incorporated into this framework as changes in the balance between bottom-up attraction forces and top-down behavioral regulation [28,29].

Taken together, these mechanisms are better understood as complementary rather than competing accounts, each describing distinct yet interacting components of a unified dynamical system underlying addictive behavior.

### 2.6. Toward a Unified Neurocomputational Perspective

Taken together, these mechanisms may be understood as components of a coupled neurocomputational system in which dopaminergic learning amplifies reward signals, salience networks prioritize selected inputs, executive systems regulate behavior, and individual variability modulates overall system sensitivity—all within environments that continuously reshape reinforcement structure.

Within this framework, behavior emerges as trajectories evolving across a dynamically changing reward landscape. This perspective shifts the explanatory focus from isolated dysfunctions toward emergent system dynamics, thereby providing a bridge between neurobiological mechanisms and large-scale behavioral organization. It also aligns with contemporary approaches in computational psychiatry, which increasingly conceptualize mental disorders as disturbances of interacting system-level processes rather than solely as localized neural deficits [25,26,27].

## 3. Reward Landscape Distortion: A Dynamical Systems Perspective on Behavioral Addiction

### 3.1. Behavioral Systems as Reward Landscapes

To better explain the emergence of behavioral addiction in digital environments, it may be useful to move beyond linear descriptions of behavior toward a state-space formulation. Within this perspective, behavior is not viewed as a sequence of isolated choices, but as a continuous trajectory evolving within a high-dimensional space of competing action possibilities [14,15,16,30].

We conceptualize this space as a reward landscape, in which each behavioral configuration corresponds to a state associated with an expected reward value. Behavioral dynamics can therefore be understood as probabilistic trajectories moving across this landscape, shaped by reinforcement learning processes and constrained by both internal neurobiological parameters and external environmental inputs [17,18,19].

Importantly, the reward landscape is used here as a theoretical construct describing probabilistic state-space organization within a formal modeling framework. It should be understood as a conceptual tool for integrating reward valuation, behavioral flexibility, and environmental structure rather than as a claim that the brain literally implements landscape-like computations or that attractor dynamics have been empirically demonstrated in digital addiction [16,19,25,26,27,30].

Conceptually, the reward landscape can be thought of as a map of how attractive different behaviors are, given their expected rewards [19,25,31]. In this view, some regions of the landscape correspond to behaviors that feel highly rewarding and therefore tend to be selected more often, functioning as attractor-like states.

Under ecologically typical conditions, reward landscapes may exhibit relatively distributed topographies, with multiple comparable peaks corresponding to diverse behavioral domains such as social interaction, learning, physical activity, and rest. Such a distributed structure may support behavioral flexibility and a broad spread of activities, which we describe informally as higher behavioral diversity rather than in strict thermodynamic terms [30,31,32].

Crucially, however, this balance is not assumed to be intrinsic to the organism alone, but may emerge from the statistical structure of environmental reinforcement and its interaction with individual neurobiological sensitivity.

### 3.2. Distortion Through Reinforcement Density and Variance

Digital environments may perturb the geometry of the reward landscape by altering two fundamental statistical properties of reinforcement: density and variance. We operationalize reinforcement density as the temporal compression of reward events and variance as the stochasticity of reward delivery—key drivers jointly determine how often value estimates are updated and how uncertain future rewards remain.

First, digital systems often increase reinforcement density. In contrast to many offline environments—where rewards may be delayed, effort-dependent, or temporally sparse—digital platforms compress reinforcement into dense temporal sequences. Notifications, continuous content streams, rapid social feedback, and low-friction access reduce the temporal distance between reward events, thereby shortening the behavioral path required for reward acquisition. This may accelerate convergence dynamics and bias behavior toward immediately accessible reward states [21,22].

Second, digital environments may amplify reward variance through stochastic and unpredictable reinforcement schedules. Variable reward structures, well established in reinforcement learning theory, can sustain dopaminergic prediction-error signaling by limiting rapid value saturation. While moderate variability may initially promote exploration [17,18,19], persistent high variance may ultimately favor selective amplification of behaviors repeatedly associated with salient or frequent rewards [20,21].

Taken together, these processes may reshape the reward landscape. Reinforcement becomes both frequent and unpredictably distributed, sustaining continuous learning while disproportionately strengthening selected behavioral pathways. As reinforcement density and variance increase, the system may shift from a distributed reward regime toward a more asymmetric configuration dominated by a limited number of behavioral states.

**Figure 2:**
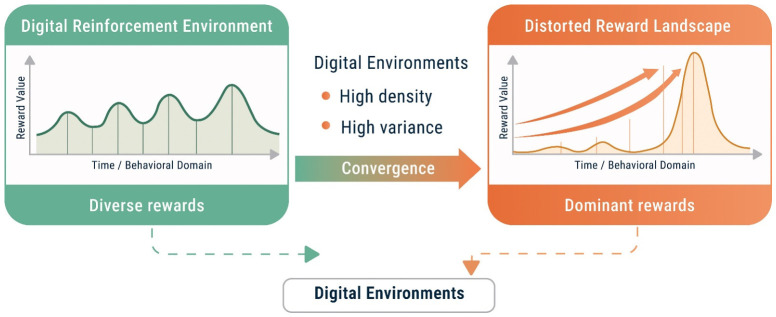
Distortion of the reward landscape under increasing reinforcement density and variance. The left panel (Digital Reinforcement Environment) depicts a distributed reward structure, in which multiple behavioral domains yield comparable reward values, supporting behavioral diversity and flexible exploration. The central arrow (Convergence) represents the transition driven by high-density and high-variance digital reinforcement. The right panel (Distorted Reward Landscape) depicts the resulting asymmetric configuration, in which a single dominant reward peak emerges, promoting convergence of behavioral trajectories toward digital engagement. The feedback loop (dashed arrows connecting both panels to the “Digital Environments” box at the **bottom**) represents a self-reinforcing cycle: as the reward landscape becomes distorted and behavior converges toward digital rewards, individuals are more likely to re-engage with the same digital platforms, thereby sustaining and amplifying high-density reinforcement exposure and contributing to persistent maladaptive engagement patterns. Solid arrows indicate directional changes in the reward landscape over time, whereas dashed arrows represent feedback dynamics between behavioral convergence and ongoing digital reinforcement.

This transformation may be understood as a reorganization of the behavioral state space, creating conditions favorable to attractor formation and progressive reduction in behavioral diversity. Figure 2 illustrates how increasing reinforcement density and variance may reshape the reward landscape from a distributed configuration toward a more skewed structure dominated by a limited number of reward peaks.

Schematic representation of how environmental reinforcement structure may reshape the topology of the reward landscape. Under low-density and low-variance conditions, the landscape exhibits a distributed configuration with multiple comparable reward peaks, supporting behavioral diversity and flexible exploration. As reinforcement density and variance increase, reward gradients may become steeper and more asymmetric, promoting the emergence of dominant attractor regions.

**Figure 3:**
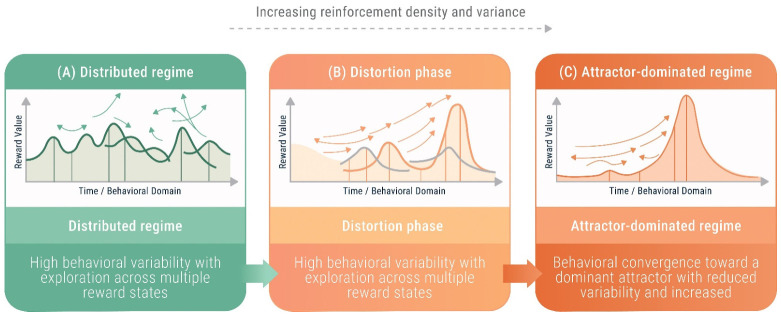
Emergence of attractor dynamics in distorted reward landscapes. Panel (**A**) depicts a distributed regime, in which the reward landscape contains multiple comparable peaks, supporting diverse behavioral trajectories and high behavioral variability. Panel (**B**) illustrates a distortion phase, in which reinforcement density and variance increase, causing partial convergence of trajectories toward emerging high-reward regions. Panel (**C**) shows an attractor-dominated regime, in which a single dominant reward peak captures most behavioral trajectories, resulting in reduced variability, increased behavioral persistence, and diminished disengagement capacity. Arrows within each panel indicate the directionality of behavioral trajectories within the evolving reward landscape. The horizontal arrow at the top represents progressive transition under sustained high-density reinforcement conditions. Return trajectories from attractor-dominated regimes (**C**) to more distributed states (**A**) remain theoretically possible but may require sustained intervention, environmental restructuring, or reduction in reinforcement density and variance.

Such structural distortion may have direct consequences for behavioral dynamics. As the landscape becomes increasingly asymmetric, behavioral trajectories may become progressively constrained, converging toward dominant regions of the landscape. This consequence is illustrated schematically in Figure 3, which depicts the transition from distributed behavioral exploration to attractor-dominated dynamics.

Schematic representation of behavioral trajectories under increasing reinforcement density and variance. (A) In a distributed regime, the reward landscape contains multiple comparable peaks, supporting diverse behavioral trajectories and high variability. (B) As reinforcement density and variance increase, the landscape becomes progressively distorted, leading to partial convergence of trajectories toward emerging high-reward regions. (C) In an attractor-dominated regime, a single dominant reward peak captures behavioral trajectories, resulting in reduced variability, increased persistence of behavior, and diminished disengagement capacity. Arrows indicate the directionality of behavioral trajectories within the evolving reward landscape.

### 3.3. Emergence of Dominant Reward Peaks

Within the proposed framework, the interaction between reinforcement density and reinforcement variance may contribute to the emergence of dominant reward peaks—regions of the landscape associated with disproportionately high expected reward values relative to competing alternatives.

Such peaks may arise through the convergence of several reinforcing factors, including high temporal frequency of reward delivery, strong attentional salience mediated by predictive cues, and low energetic or cognitive cost of engagement. When these factors co-occur, the expected reward associated with specific behaviors may become substantially elevated compared with alternative activities that require greater effort, delay, or sustained commitment [21,22].

This growing asymmetry may produce a steepening of reward gradients, such that even small deviations toward high-reward behaviors increase the probability of rapid convergence into their corresponding basins of attraction. In practical terms, behaviors offering immediate, salient, and repeatedly accessible rewards may become progressively easier to re-enter and more difficult to disengage from.

As a consequence, the reward landscape may become increasingly biased, channeling behavioral trajectories toward a narrower subset of highly reinforced states while reducing engagement with more distributed or delayed reward sources. Such dynamics may help explain how repetitive digital behaviors become progressively dominant over time.

### 3.4. Collapse of Behavioral Entropy

A potential consequence of increasing reward asymmetry is the progressive reduction in behavioral entropy, defined here as the diversity and dispersion of action selection across available behavioral options. In the present context, entropy is used in an information-theoretic or behavioral diversity sense rather than as a strict thermodynamic measure.

Behavioral entropy has been linked to cognitive flexibility and adaptive system dynamics, with lower entropy associated with more constrained behavioral repertoires and diminished exploratory capacity in both neural and behavioral systems [30,31,32].

In relatively distributed reward landscapes, behavioral entropy may remain comparatively high, supporting flexible transitions between activities and broader allocation of attention and effort. However, as dominant reward peaks emerge, action selection may become increasingly concentrated around a narrower set of rewarding behaviors.

This transition can be interpreted as a shift from exploration toward exploitation. Behavioral variability may function as a stabilizing property of the system; its erosion can reduce the capacity to explore alternative regions of the landscape and adapt to changing contingencies.

As behavioral entropy declines, the system may become increasingly susceptible to attractor formation, repetitive behavioral patterns, and reduced disengagement flexibility.

### 3.5. Attractor Formation and Phase Transition Dynamics

Terminology and empirical status: In this section, terms such as “attractor states,” “phase transitions” are used as theoretical organizing concepts borrowed from mathematical models of complex systems [14,15,16,19]. These terms do not imply that such dynamics have been empirically verified in digital addiction, nor that the brain implements formal attractor mechanisms as described in physics or computational neuroscience. Instead, they offer a simple way to think about how certain behavioral patterns can emerge, persist, and become hard to change under specific reinforcement conditions. Empirical validation of these proposed dynamics requires longitudinal studies capable of detecting non-linear transitions, critical slowing down, and other predicted signatures—work that remains to be conducted [19,22,23,24].

Within a dynamical systems framework, the processes described above may culminate in the emergence of relatively stable attractor-like states toward which behavioral trajectories converge and from which disengagement becomes progressively less likely. In this context, repeated behavioral selection may increase the likelihood of future re-selection, thereby strengthening persistence over time.

We propose that behavioral addiction may, in some cases, resemble a phase-transition-like reorganization in the structure of the reward landscape. Below a critical threshold, the system may remain in a multi-stable regime, with multiple competing attractors supporting behavioral flexibility and shifting patterns of engagement [14,15,30]. Above this threshold, the system may undergo a qualitative reorganization toward a dominant attractor characterized by:

- high entry probability,
- reduced exit probability,
- diminished sensitivity to alternative rewards.

Such a transition is hypothesized to be shaped by the interaction between intrinsic reward sensitivity, environmental reinforcement density and variance, and behavioral variability. Importantly, these dynamics are expected to be non-linear, such that relatively small parameter changes may produce disproportionately large behavioral effects. This interpretation is broadly consistent with contemporary models of dynamical systems, tipping-point behavior, and neural state transitions [10,24].

Although presented here as a conceptual framework rather than a proven empirical law, this perspective may help explain why some individuals show abrupt shifts from flexible engagement to persistent, difficult-to-reverse behavioral patterns under sustained digital reinforcement conditions. These proposed dynamics remain hypothetical and require empirical testing using longitudinal behavioral, neurocognitive, and digital phenotyping data capable of capturing transitions in system stability over time.

### 3.6. Irreversibility and Path Dependence

Once established, attractor-like states may exhibit substantial path dependence, meaning that prior behavioral trajectories can influence subsequent system evolution [14,19,30]. Repeated reinforcement may progressively deepen the attractor basin, increasing persistence and reducing responsiveness to competing alternatives or external perturbations.

This perspective offers a potential mechanistic explanation for a common clinical observation: individuals may continue maladaptive behavioral patterns despite explicit awareness of negative consequences [4,10,28,29]. Within the present framework, such persistence is interpreted not solely as a failure of volitional control, but as a property of system organization in which previously reinforced trajectories become increasingly dominant over time.

As this process unfolds, alternative behavioral pathways may remain available in principle, yet become progressively less accessible in practice because they carry lower immediate reward value, require greater effort, or lack sufficient reinforcement history. This formulation may help explain why behavioral change often requires sustained restructuring of environmental contingencies rather than simple intention alone.

### 3.7. Synthesis: Addiction as an Emergent Property of Distorted Landscapes

The framework developed here reconceptualizes behavioral addiction as an emergent property of a coupled brain–environment system whose reward landscape may become progressively distorted over time. Rather than arising from a single cause, maladaptive behavioral persistence is viewed as the product of interacting neurobiological, behavioral, and environmental processes.

Within this perspective, neurobiological mechanisms govern learning, valuation, and plasticity, while environmental structures shape the density, variance, and accessibility of reinforcement. Under sustained high-density and high-variance reinforcement, the system may become increasingly biased toward dominant reward peaks, thereby increasing the likelihood of stable attractor-like states.

From a systems perspective, this transition may also be interpreted as a reduction in behavioral entropy, reflecting constrained exploration and reduced diversity of action selection within the available state space [30,31,32].

Accordingly, addiction is not reduced to a single mechanism or localized dysfunction, but conceptualized as a system-level reorganization of motivational dynamics driven by the interaction between neural learning processes and altered environmental reward structures.

## 4. Behavioral Reward Instability Index (BRII): A Heuristic Framework for Motivational Instability

### 4.1. Conceptual Rationale

To move beyond purely qualitative description toward a testable systems model, it is helpful to introduce a single summary variable that captures how the system is evolving within this landscape [11,12,13,25,26,27]. In this context, the BRII is not introduced as a new psychometric scale, but as a heuristic systems construct inspired by existing approaches in computational psychiatry, control theory, and dynamical systems modeling.

We therefore propose the Behavioral Reward Instability Index (BRII) as a heuristic system-level construct describing the degree to which a behavioral system may approach a regime of instability characterized by attractor dominance and reduced behavioral flexibility. Such formulations are broadly consistent with contemporary approaches in computational psychiatry and dynamical systems neuroscience, which often emphasize interacting state variables rather than isolated behavioral measures [25,26,27].

Importantly, the BRII is not intended as a diagnostic score, clinical label, or directly validated scalar measure. Rather, it is conceptualized as a latent systems variable reflecting the interaction between individual reward sensitivity, environmental reinforcement structure, and emergent behavioral organization [11,12,13].

In this sense, the BRII serves as a compact representation of system dynamics, capturing interactions that would otherwise require multidimensional behavioral description. Comparable approaches have been used in models of large-scale neural and behavioral dynamics, where complex processes are approximated through lower-dimensional control parameters linked to regime shifts or changes in stability [30,31,32].

Within the present framework, the BRII is proposed as an indicator of proximity to transitions between behavioral regimes, ranging from relatively distributed reward engagement to patterns increasingly dominated by a limited number of highly reinforced attractor-like states. Terms such as attractor-like states are used in an operational systems sense to denote relatively persistent behavioral configurations, without assuming strict mathematical attractors have yet been demonstrated empirically. At present, the BRII should be regarded as a hypothesis-generating framework rather than a validated measurement instrument.

### 4.2. Core Dimensions

Within the present framework, the BRII is conceptualized as emerging from the interaction of three coupled dimensions operating at distinct but interrelated levels of analysis: individual reward sensitivity, digital reward exposure, and behavioral variability. These three dimensions are intended as parsimonious core domains rather than an exhaustive ontology of motivational determinants; additional modulators such as stress, affective state, sleep disruption, or social context may be incorporated in future model extensions.

#### 4.2.1. Individual Reward Sensitivity (IRS)

This dimension reflects variability in responsiveness to rewarding stimuli, shaped by genetic, neurobiological, and trait-related factors such as dopaminergic responsivity, impulsivity, delay discounting tendencies, and executive regulation. Such factors may modulate the effective gain of reinforcement learning processes and influence how strongly reward differences affect behavior. Variability in reward sensitivity has been associated with differences in reward learning, impulsivity, and addiction vulnerability [6,21,22,28,29]. IRS should be interpreted as a higher-order latent sensitivity construct rather than a single biological trait, potentially decomposable into separable subcomponents in future empirical work.

#### 4.2.2. Digital Reward Exposure (DRE)

This dimension captures the density, immediacy, and stochastic structure of reinforcement signals in digital environments, including the frequency, timing, and unpredictability of reward delivery. Reinforcement learning research suggests that both reward density and variance can substantially influence learning dynamics, cue salience, and value updating [17,18,19].

#### 4.2.3. Behavioral Variability (BV)

This dimension reflects the diversity and dispersion of behavioral engagement across activities. It is conceptualized as a stabilizing influence that may counteract convergence toward a single dominant behavioral state. Behavioral entropy and variability have been linked to cognitive flexibility and adaptive system dynamics in both neural and behavioral domains [25,26,30,31,32].

Together, these components define a coupled system in which individual reward sensitivity modulates responsiveness to reinforcement, digital reward exposure shapes environmental input intensity and variability, and behavioral variability influences resilience through distributed engagement. Crucially, instability is hypothesized to emerge from the interaction of these components rather than from any single factor in isolation. This interaction-based perspective is consistent with contemporary models of addiction and decision-making that emphasize non-linear interplay between biological predispositions and environmental inputs [8,9,10].

The framework also naturally extends to a temporal perspective, in which instability may evolve dynamically over time. This temporal form is denoted as BRII(t), where the index is evaluated at successive time points or across sliding observation windows. From this viewpoint, repeated longitudinal assessment could examine whether early warning signals precede regime shifts, whether destabilization and recovery follow asymmetric trajectories, and whether some individuals exhibit hysteresis-like persistence after environmental conditions improve. Such patterns would be broadly consistent with critical slowing down and tipping-point dynamics described in other complex systems. Temporal dynamics of BRII are summarized in Table 2 [14,19,22,23,24].

### 4.3. Heuristic Non-Linear Formulation

A defining property of reward instability is its proposed non-linear nature. Behavioral systems exposed to high-density reinforcement may not change proportionally; instead, relatively small parameter shifts may produce disproportionately large behavioral effects. Comparable amplification dynamics have been described in reinforcement learning, neural adaptation, and other complex systems [14,18,19,30].

To represent this concept heuristically, the BRII may be expressed in simplified form as:

BRII ∝ (IRS × DRE)/BV

where ∝ denotes “is proportional to,” meaning that BRII becomes higher when reward sensitivity (IRS) and digital reward exposure (DRE) increase, and becomes lower when behavioral variability (BV) is greater [14,15,16,17,18,20]. All components are assumed to be normalized for conceptual clarity. We use the proportionality symbol (∝) rather than an equality sign (=) to indicate that this expression is a conceptual guide to the direction of effects, not a fixed mathematical law. For practical modeling purposes, an approximate equality version is provided in the Supplementary Materials (Section S1.3), where BRII(t) is calculated from normalized parameters using a simple numerical adjustment.

The proposed formulation reflects three core principles. First, multiplicative amplification: higher individual reward sensitivity (IRS) may increase responsiveness to environmental reinforcement (DRE), thereby enhancing reward-driven learning. Second, environmental driving force: reinforcement density and variance may continuously bias the system toward convergence on salient reward states. Third, stabilizing variability: behavioral variability (BV) distributes engagement across multiple domains, potentially counteracting excessive convergence and supporting flexibility.

The multiplicative structure further implies that identical environmental inputs may produce different outcomes depending on system configuration. For example, the impact of digital reward exposure may increase under higher reward sensitivity, whereas lower behavioral variability may amplify vulnerability to reinforcement-driven convergence. Such interaction effects are consistent with contemporary models of addiction in which risk emerges from coupled biological and environmental influences rather than from any single factor alone [8,9,10].

Accordingly, instability is hypothesized to arise when amplification processes outweigh stabilizing influences, increasing the likelihood of progressive convergence toward dominant attractor-like states. Alternative mathematical forms (for example, threshold, sigmoid, or exponential functions) may ultimately describe these dynamics more accurately and would need to be calibrated with empirical data. At present, the BRII is best understood as a latent systems construct rather than a directly measurable score. Any future operational version would need clear rules for normalization, scaling, and domain-specific calibration before its numerical values could be meaningfully interpreted.

### 4.4. Operationalization Using Digital Phenotyping

While the BRII is introduced as a conceptual systems variable, provisional operational approximations may be developed for exploratory modeling purposes, particularly using multimodal data derived from digital phenotyping [23,24]. In this context, the BRII is not assumed to be directly observable; instead, its component processes may be indirectly approximated through behavioral and contextual proxies such as app-switching entropy, notification density, or recovery-from-perturbation metrics. Crucially, these indicators are currently best regarded as provisional, hypothesis-generating proxies that lack established construct validity in digital addiction research and therefore require dedicated validation studies before they can support strong interpretive or clinical claims. Potential examples are summarized in Table 2.

The BRII is conceptualized as a latent system-level construct emerging from the interaction between individual reward sensitivity, environmental reinforcement structure, and behavioral variability. Accordingly, its dimensions may be approximated using passive and active longitudinal data streams generated by smartphones, wearable devices, and digital behavioral tasks [23,24]. Such approaches may enable preliminary modeling of reward-system dynamics in real-world settings.

Examples of candidate indicators include impulsivity indices, delay discounting performance, and neurocognitive task measures as proxies of individual reward sensitivity; screen time, notification frequency, short-form content exposure, and app engagement patterns as indicators of digital reward exposure; and behavioral entropy, activity diversity, sleep regularity, mobility patterns, or app-use diversity as markers of behavioral variability. Repeated measures may also permit estimation of temporal dynamics, including recovery from perturbation, increasing variance, or slowing return to baseline following behavioral disruption.

These data sources offer a scalable—although indirect—empirical substrate for testing the present framework. However, important limitations must be acknowledged. Digital phenotyping signals are inherently noisy, context-dependent, and sensitive to device usage patterns, platform design, and missing data. Common behavioral proxies may capture only part of the underlying motivational processes, and measurement bias may differ across populations and operating systems [23,24].

Consequently, any proxy-based operationalization of the BRII should be interpreted as a structured approximation of system dynamics rather than a direct measurement of latent constructs; proxy indicators should therefore be interpreted probabilistically and at the aggregate trajectory level rather than as precise readouts of latent reward-system states. Robust validation will require longitudinal studies combining behavioral data, neurocognitive assessment, and external clinical outcomes. Any future operational BRII scores should not be used for individual-level clinical decision-making without robust prospective validation.

### 4.5. Limits and Future Validation

The BRII is not intended to diagnose addiction, replace clinical assessment, or define pathological thresholds in its current form. Rather, it is intended as a conceptual bridge between the theoretical framework of reward landscape dynamics and empirical observation. By representing instability as a system-level construct emerging from interacting components, it may help formalize processes that are otherwise described qualitatively [25,26,27].

At present, however, the BRII remains a hypothesis-generating framework rather than a validated measurement instrument. The framework should also be considered falsifiable, insofar as longitudinal data may fail to support predicted instability dynamics or incremental predictive utility. Its components require empirical calibration, operational definitions may vary across settings, and causal interpretations should be made cautiously. Future research should test whether BRII-informed models improve prediction of behavioral persistence, relapse risk, or recovery trajectories beyond conventional single-metric indicators [11,12,13,23,24].

Validation will likely require prospective longitudinal studies integrating passive digital sensing, neurocognitive measures, ecological momentary assessment, and clinically meaningful outcomes.

If supported empirically, the BRII may offer a useful translational framework for identifying periods of elevated motivational instability and informing adaptive intervention strategies. Future comparisons should explicitly evaluate whether BRII-informed models add incremental explanatory or predictive value beyond conventional indicators such as screen time, impulsivity scores, or symptom counts. Although inspired by digital environments, the present framework may require substantial modification before extension to substance-related or non-digital behavioral addictions.

A proof-of-concept translational extension illustrating how BRII states may inform structured behavioral interpretation and intervention logic is provided in Supplementary Material S1.

## 5. Toward Operationalization: Digital Phenotyping and Reward Instability

### 5.1. Digital Phenotyping as an Empirical Substrate

The Reward Instability framework conceptualizes behavioral addiction as a system-level phenomenon emerging from interactions between individual reward sensitivity, environmental reinforcement structure, and behavioral dynamics. Although the BRII is proposed as a conceptual systems construct, its dimensions may be indirectly approximated through observable behavioral proxies.

Digital phenotyping—defined as the continuous quantification of behavior using passive and active data streams from personal digital devices—offers a potential empirical substrate for examining such dynamics [23,24]. Smartphones and wearable technologies can generate longitudinal data reflecting engagement patterns, attentional allocation, mobility, sleep regularity, and activity distribution across time [23,24].

Candidate proxies for core BRII dimensions are summarized in Table 2. Repeated measurement may additionally support assessment of temporal instability, persistence, and recovery dynamics.

Importantly, these indicators do not directly measure latent motivational constructs. They should therefore be interpreted as indirect signals approximating underlying system dynamics rather than as direct measurements.

### 5.2. Measurement Challenges: Noise, Validity, Bias, and Governance

Despite its promise, digital phenotyping also introduces substantial methodological constraints that must be considered when evaluating the BRII framework.

First, signal noise represents a fundamental limitation. Behavioral data collected from digital devices are often incomplete, context-dependent, and influenced by measurement artifacts. Variability in device usage, operating systems, platform design, and data resolution may obscure the underlying dynamics of interest.

Second, construct and ecological validity remain limited. Many commonly used indicators—such as screen time or app frequency—serve only as coarse proxies for complex motivational processes. High engagement does not necessarily imply reward dominance, and low engagement does not guarantee behavioral stability. The same observable behavior may reflect boredom, work demands, social connection, or maladaptive reinforcement, depending on context.

Third, sampling and systemic bias may arise from uneven data representation. Digital phenotyping datasets are often skewed toward particular demographic groups, socioeconomic strata, device ecosystems, and cultural settings. In addition, observed behavior is partly shaped by algorithmic curation and platform design, complicating causal interpretation [23,24,33,34,35].

Fourth, privacy, consent, and governance considerations are central. Continuous behavioral monitoring raises important ethical questions regarding informed consent, data security, transparency, and acceptable downstream use of inferred risk states.

Taken together, these limitations imply that any empirical implementation of the BRII should be interpreted as a structured approximation of system dynamics rather than a direct measurement of latent processes. Similar concerns have been highlighted in recent work on digital phenotyping and computational psychiatry [23,24].

To illustrate how the BRII framework could identify population-level trends in problematic digital engagement, we present a simulated dataset of 100 hypothetical digital users with varying reward sensitivity, digital reinforcement exposure, and behavioral diversity profiles (see Supplementary Materials, Section S1.12, Figure S2) [19,20,23,24]. This simulation demonstrates that individuals with elevated BRII scores—reflecting the combination of high reward sensitivity, high-density digital reinforcement, and reduced behavioral variability—cluster in a manner consistent with problematic engagement patterns. Importantly, this example illustrates the BRII’s intended use as a research tool for identifying vulnerability trends at the population level, rather than as a diagnostic instrument for individual classification [23,24,33].

### 5.3. Non-Linearity and Calibration Requirements

A key implication of the Reward Instability framework is that the relationship between observable indicators and underlying system dynamics may be inherently non-linear. Small changes in reinforcement density, environmental cues, or behavioral variability may produce disproportionately large effects depending on baseline system configuration. Consequently, simple linear aggregation of behavioral metrics may fail to detect critical transitions in motivational state. Such non-linear effects are well described in reinforcement learning and dynamical systems models of behavior [19,24,34].

Future empirical work should therefore prioritize:

- non-linear modeling approaches (e.g., multiplicative, threshold-based, or sigmoid formulations),
- dynamic time-series analyses capturing trajectory evolution over time,
- person-specific baselines and within-subject change detection [23,24],
- identification of thresholds or early warning signals associated with attractor formation.

Meaningful calibration of the BRII would likely require longitudinal datasets capable of capturing transitions between relatively stable and unstable behavioral regimes.

### 5.4. Scope and Limits of Operationalization

It is important to emphasize that the primary contribution of the present work is theoretical rather than operational. The proposed framework is not intended as a finalized measurement system, validated clinical tool, or instrument for immediate decision-making. Rather, it is offered as a conceptual structure for understanding reward-driven behavioral dynamics.

Accordingly, empirical approximation through digital phenotyping should be viewed as a secondary and exploratory extension intended to test, refine, and potentially falsify the theoretical model. At present, the framework is best regarded as hypothesis-generating rather than decision-guiding.

### 5.5. Synthesis: Measurement as Model Refinement

Digital phenotyping offers a potential pathway for linking theoretical constructs with empirical observation. Its primary value, however, may lie less in precise measurement than in enabling iterative refinement of systems-level models.

Within the present framework, empirical data may be used to:

- test whether non-linear transitions occur in real-world behavioral dynamics,
- identify conditions under which reward landscapes become progressively distorted,
- refine the structure, calibration, and predictive utility of the BRII framework.

By maintaining a clear distinction between conceptual modeling and proxy-based approximation, the present approach avoids reducing complex motivational dynamics to simplified single-metric interpretations.

## 6. Discussion

### 6.1. From Mechanisms to System Dynamics

The present framework proposes a shift from mechanism-centered accounts of behavioral addiction toward a systems-level understanding of motivational dynamics. Rather than attributing maladaptive behavior to isolated neural dysfunctions, Reward Instability Theory conceptualizes addiction as an emergent property of a coupled brain–environment system in which reward landscapes may become progressively distorted over time.

Within this perspective, outcomes are defined less by any single behavior and more by the dynamical state of the system—namely, whether behavioral trajectories remain relatively distributed and flexible or increasingly converge toward dominant attractor-like patterns. This distinction may help explain why superficially similar behaviors can differ substantially in persistence, reversibility, and functional impact.

Accordingly, the present work shifts the unit of analysis from discrete behavioral acts to system dynamics, broadly aligning with contemporary directions in computational psychiatry that emphasize interacting state variables over isolated symptoms or static diagnostic categories [11,12,13].

### 6.2. Unique Theoretical Contribution and Testable Predictions

The Reward Instability framework builds upon established neuroscience and addiction models, but introduces three specific advances that extend beyond re-labeling or integrating existing concepts.

First, environmental primacy in system organization. Unlike traditional addiction models that primarily attribute risk to individual vulnerabilities (e.g., dopaminergic sensitivity, impulsivity, executive dysfunction), the present framework assigns equal explanatory weight to environmental reinforcement structure as a direct driver of behavioral dynamics. Specifically, we propose that reinforcement density and variance—properties of digital platforms rather than individuals—can actively reshape the reward landscape’s topology, thereby altering behavioral trajectories independently of baseline neurobiological state [20,35,36]. Current addiction models typically treat environmental factors as contextual modifiers rather than as primary determinants of system-level reorganization [3,4,20]. By contrast, the present framework predicts that identical individuals may exhibit substantially different behavioral outcomes depending on the statistical structure of reinforcement exposure—a prediction directly testable through platform-manipulation experiments or cross-platform comparisons [23,24,35,36].

Second, prediction of non-linear transitions and early warning signals. The framework generates testable predictions that existing models do not explicitly address. We predict that (i) behavioral systems may exhibit non-linear transitions rather than gradual linear deterioration, (ii) increased variance and slowed recovery from perturbation may precede overt behavioral consolidation (analogous to critical slowing down in dynamical systems) [19,20,22], and (iii) reversibility may depend on attractor depth and duration of exposure rather than symptom count alone. Concrete operationalization strategies and study designs are described in Section 6.3. Crucially, these dynamics are not reducible to existing constructs such as tolerance, withdrawal, or craving—they describe system-level properties of behavioral trajectories that emerge from interacting components [11,14,15,16,25,26,27].

Third, integrative architecture unifying mechanism-level theories. Rather than proposing another isolated mechanism, the framework provides a shared dynamical structure within which incentive sensitization (steepening of reward gradients [5]), habit formation (attractor stabilization [4,20]), and allostatic dysregulation (landscape deformation [10,20]) can be interpreted as complementary processes operating at different levels of system organization (Section 2.5). This integration shifts the unit of analysis from discrete mechanisms toward system-level trajectories evolving within a structured state space [11,12,13,14,15,16,25,26,27].

We have clarified these contributions in Section 6.2 (Theoretical Contribution and Testable Predictions) and Section 1 (Introduction). We also emphasize that the framework is hypothesis-generating and requires empirical validation through longitudinal studies capable of capturing behavioral dynamics over time [19,22,23,24]. In summary, the framework’s innovation lies in environmental emphasis and systems-level integration: it explains how engineered digital environments may reorganize behavior through reward landscape distortion, generating testable predictions regarding temporal precedence, non-linear thresholds, reversibility, and cross-platform generalizability (detailed in Section 6.3).

### 6.3. Empirical Operationalization and Testable Predictions

While the BRII is formalized as a systems-level construct, it can be empirically approximated through proxies derived from behavioral time-series data and self-report measures. We propose the following operational framework.

#### 6.3.1. Operationalization of BRII Components

Reward sensitivity (α) may be estimated via self-reported reward responsivity scales (e.g., BAS-Drive subscale [29]) or behavioral proxies such as app-switching rates following notification receipt. Environmental reinforcement structure can be quantified through digital phenotyping metrics: reinforcement density (ρ) operationalized as notification frequency or interaction rate, and reinforcement variance (σ^2^) measured as variability in engagement duration or reward magnitude across sessions. Behavioral variance (Var[b]) may be captured as entropy of app-switching patterns or inverse concentration indices (e.g., Herfindahl index) reflecting diversity of digital activities [23,24,30,31,32]. These proxies enable time-resolved estimation of BRII dynamics without requiring direct neural measurement. Importantly, IRS, DRE, and BV are conceptualized as latent constructs inferred from observable indicators: IRS primarily reflects trait-level reward sensitivity (with state-level variation), DRE is a time-varying environmental parameter, and BV is a dynamic behavioral outcome. These dimensions are theoretically separable (individual, environmental, and emergent levels) but empirically interactive, requiring multi-level study designs for proper disentanglement [11,25,26,27].

#### 6.3.2. Proposed Study Designs

Three complementary empirical approaches could test core predictions. First, longitudinal digital phenotyping studies tracking passive smartphone usage in N = 200–500 participants over 6–12 months, paired with bi-weekly self-report assessments, could test whether increases in reinforcement density precede reductions in behavioral diversity, and whether baseline reward sensitivity moderates this trajectory [23,24]. Second, experimental manipulation designs could assign participants to modified social media environments with controlled notification schedules (high vs. low density; fixed vs. variable intervals) to test whether high-density variable reinforcement produces greater attractor-like convergence (measured as reduced exploration of alternative activities) and whether effects are moderated by individual differences [17,18,19,35,36]. Third, computational agent-based simulations implementing BRII parameters (α, ρ, σ^2^, Var[b]) could model behavioral trajectories under varying reinforcement landscapes and validate whether simulated attractors reproduce clustering patterns observed in real-world digital phenotyping data [17,18,19,30,34].

#### 6.3.3. Falsifiable Predictions

The framework generates four testable predictions with explicit falsification criteria. First, temporal precedence: increases in reinforcement density should precede (not follow) reductions in behavioral diversity; reverse causality would challenge the landscape distortion hypothesis. Second, dose–response relationship: BRII estimates should show monotonic association with clinical symptom severity; non-monotonic or null relationships would suggest lack of predictive validity. Third, experimental reversibility: interventions reducing reinforcement density (e.g., notification limits) should restore behavioral variance; failure to observe recovery would indicate dominance of mechanisms beyond reinforcement structure. Fourth, cross-platform generalizability: BRII dynamics should generalize across digital environments (social media, gaming, streaming); platform-specific effects inconsistent with reinforcement structure would necessitate model revision [19,20,23,24].

#### 6.3.4. Methodological Constraints

Important challenges remain. Passive digital phenotyping offers naturalistic data but introduces noise from unmeasured confounds (mood fluctuations, social context), while experimental designs provide control at potential cost to ecological relevance [23,24]. BRII parameters likely vary across individuals and time, requiring mixed-effects modeling or idiographic approaches to capture person-specific dynamics [25,26,27]. Observational designs cannot definitively establish causality; microrandomized trials (e.g., Just-In-Time Adaptive Interventions) may offer stronger causal leverage by randomizing reinforcement exposure at the momentary level [23,24,30,31,32]. These constraints underscore that the framework remains hypothesis-generating and requires systematic empirical validation.

An additional constraint concerns the nonspecificity of proposed measurement proxies. Metrics such as screen time, app diversity, and notification frequency are indirect indicators that may reflect multiple underlying processes—including adaptive high engagement, context-appropriate use, or external demands—not exclusively problematic patterns. These proxies lack construct specificity and require validation against clinically meaningful outcomes (e.g., functional impairment, distress, behavioral rigidity) to establish discriminant validity [3,20,23,24]. Future empirical work must demonstrate that these measures reliably differentiate problematic from normative engagement patterns.

Moreover, the psychometric properties of BRII—including reliability, temporal stability, dimensionality, discriminant validity, and measurement invariance across populations—remain entirely unestablished. It is unclear whether BRII should be interpreted as a stable trait-like index (between-subject comparisons) or a dynamic state variable (within-subject trajectories), and whether the measure exhibits sufficient test–retest reliability or sensitivity to change. Establishing these properties will require dedicated validation studies with repeated assessments, diverse samples, and convergent-discriminant validity testing against established clinical measures [23,24,25,26,27].

Given these substantial methodological uncertainties, BRII should be treated with considerable caution. At present, it functions as a hypothesis-generating research heuristic rather than a validated clinical tool. It is not suitable for individual diagnosis, clinical decision-making, or population screening until rigorous psychometric validation, cross-validation across independent samples, and demonstration of clinical utility have been completed. Any claims regarding early warning signals or vulnerability detection remain speculative and require systematic empirical substantiation [19,22,23,24].

### 6.4. Integration Across Levels of Theory and Early Warning Signals

As discussed in Section 2.5, the framework integrates established addiction models—incentive sensitization, habit formation, and allostatic dysregulation—as complementary transformations within a shared dynamical architecture [4,5,10,20]. Throughout the manuscript, dynamical systems terminology (attractors, phase transitions, critical slowing down) is used as a theoretical heuristic for describing relative persistence, reduced behavioral flexibility, and potential non-linear transitions—not as empirically demonstrated properties of digital addiction or as claims that the brain implements formal attractor dynamics [11,14,15,16,19,25,26,27].

An additional implication of this framework is that observable changes in behavioral dynamics may precede overt consolidation of maladaptive patterns. Rather than assuming abrupt or binary transitions, systems may exhibit gradual shifts in variability, persistence, and responsiveness as they approach regimes characterized by reduced flexibility.

In this context, increased fluctuations in behavior, slower recovery following perturbation, or progressive narrowing of behavioral diversity may be broadly consistent with dynamics described in other complex systems, including critical slowing down and path dependence [14,19]. Importantly, the presence of such patterns in digital addiction has not been empirically demonstrated; these are theoretical predictions requiring longitudinal validation. Such patterns should not be interpreted as definitive markers of impending transition, but as provisional indicators of changing system stability if empirically observed.

### 6.5. Digital Environments and Measurement Implications

A key implication is the reconceptualization of digital environments as active reward-shaping systems [17,18,19,35,36] that continuously modulate reinforcement structure (Section 1 and Section 3). As a result, behavioral outcomes may not be fully understood independently of the environments in which they emerge. Importantly, not all highly engaging digital behavior should be interpreted as pathological; adaptive, recreational, educational, and socially meaningful engagement may occur within the same environments.

Importantly, the reconceptualization of digital environments as reward-shaping systems does not imply that neurobiological mechanisms alone determine behavioral outcomes. Psychosocial and contextual factors—including loneliness, peer influence, developmental stage, economic pressures, and cultural norms—may substantially moderate how individuals respond to high-density reinforcement structures [3,4,5,20,28,29]. For example, adolescents experiencing social isolation may exhibit heightened sensitivity to digital social rewards, effectively lowering the threshold for attractor formation within platforms offering peer validation [21,28,29]. Similarly, cultural contexts that normalize persistent digital connectivity or economic systems that incentivize content creation may amplify reinforcement density at the environmental level [35,36]. The present framework does not replace psychosocial accounts of digital behavior; rather, it provides a systems architecture within which neurobiological, psychological, and contextual influences may interact to shape reward landscape dynamics [11,14,15,16,25,26,27]. Understanding these multi-level interactions represents a critical direction for future empirical and theoretical work.

A related clarification concerns the distinction between high engagement and pathological persistence. The framework is not intended to pathologize frequent digital use per se. Many individuals engage extensively with digital platforms in ways that remain volitional, contextually flexible, and consistent with broader life goals [3,20]. The present model is specifically concerned with explaining the emergence of attractor-like dynamics characterized by (i) reduced behavioral flexibility, (ii) persistence despite awareness of negative consequences, and (iii) diminished capacity for disengagement [4,5,20,21]. These features distinguish problematic engagement from normative high-frequency use. Future empirical work should operationalize these distinctions using measures of behavioral rigidity, context-insensitivity, and functional impairment alongside usage frequency [23,24].

Boundary conditions and protective mechanisms: It is important to note that high reinforcement density does not invariably produce maladaptive convergence. Many adaptive behavioral domains—including learning, creativity, professional engagement, and meaningful social connection—also involve high-frequency reinforcement, persistent engagement, and attentional salience [7,17,18]. What differentiates adaptive from maladaptive high-density reinforcement likely depends on several boundary conditions: (i) behavioral flexibility—whether engagement remains responsive to contextual demands and alternative goals; (ii) functional alignment—whether the behavior serves broader life goals rather than displacing them; (iii) psychological autonomy—whether engagement is experienced as volitional rather than compulsive; and (iv) reward structure diversity—whether reinforcement is distributed across multiple meaningful domains rather than concentrated in a single behavioral sink [4,5,20,28,29]. Protective mechanisms may include metacognitive awareness, supportive social environments that encourage behavioral diversity, and platform design features that promote rather than undermine autonomy [23,24,35,36]. Future work should empirically identify the specific conditions under which high-density reinforcement supports adaptive persistence versus maladaptive rigidity.

Within this perspective, the BRII offers a conceptual pathway for translating reward instability into empirical research. However, any operationalization remains indirect. Specific measurement approaches and analytic strategies are detailed in Section 6.3. If supported empirically, such approaches may help identify periods of elevated vulnerability and guide timing-sensitive behavioral interventions [19,22,23,24].

### 6.6. Limitations

First, the framework relies on dynamical systems terminology—including attractor states, phase transitions, reward landscapes, and critical slowing down—as conceptual organizing constructs [14,15,16,19]. These terms are used heuristically to describe hypothesized behavioral dynamics and do not imply that such processes have been empirically verified in digital addiction contexts [11,25,26,27]. Formal validation would require longitudinal studies with sufficient temporal resolution to detect non-linear transitions, establish attractor-like properties, or identify early warning signals [19,22,23,24]. Furthermore, the framework does not yet provide formal mathematical specifications for key constructs: the dimensions of the reward landscape, precise definitions of state variables, operational criteria for identifying attractors, or the specific type of entropy (e.g., Shannon entropy vs. behavioral dispersion) [16,30,31,32]. Similarly, observable criteria for detecting phase transitions remain to be empirically defined. These represent important directions for future formalization and computational modeling work [11,25,26,27].

Second, the manuscript uses terms such as ‘problematic digital engagement,’ ‘behavioral addiction,’ and ‘digital addiction’ to describe a spectrum of dysregulated patterns. While we provide conceptual clarification in Section 1, the framework does not yet offer precise operational criteria for distinguishing pathological persistence from normative high engagement. Such boundaries likely depend on context-specific thresholds of behavioral rigidity, functional impairment, and subjective distress—dimensions that require empirical calibration and may vary across individuals, developmental stages, and cultural contexts [3,4,5,20,28,29].

Third, the framework is primarily conceptual and does not yet constitute a fully specified quantitative model. Fourth, constructs such as reward landscapes, attractor states, and instability indices are abstractions that simplify substantial biological and behavioral complexity. Fifth, digital phenotyping approaches introduce methodological constraints, including measurement noise, limited ecological validity, sampling bias, and privacy concerns [23,24].

Sixth, the present formulation focuses primarily on reward-system mechanisms and provides limited integration of broader psychosocial, developmental, and cultural determinants. Factors such as loneliness, social identity, peer dynamics, developmental stage, economic incentives, and cultural variation may substantially shape both individual vulnerability and environmental reinforcement structure [3,4,5,20,28,29,35,36]. Although these influences can be conceptualized as modulators of reward landscape topology (Section 2.4), the framework does not yet provide a formal account of how social context interacts with neurobiological parameters to produce observed behavioral variability [11,14,15,16,25,26,27]. Addressing this limitation will require empirical studies capable of capturing individual-level psychosocial variables alongside high-resolution behavioral and environmental data, as well as theoretical extensions that explicitly model social and contextual processes within the dynamical systems architecture [23,24]. Finally, the model has been developed with particular emphasis on high-density digital environments and may not generalize directly to other behavioral domains without modification [20,35,36].

### 6.7. Future Directions

The present framework nevertheless suggests several directions for future research. From a modeling perspective, integrating reinforcement learning with dynamical systems approaches may enable more formal representations of how reward landscapes evolve over time [17,18,19,34]. More broadly, the framework highlights a potential multi-scale temporal structure in which fast fluctuations in reinforcement signals interact with slower processes of learning, adaptation, and self-regulation [14,19,30,31,32]. Understanding these cross-timescale interactions may help explain why some behavioral patterns become resistant to change despite conscious awareness.

## 7. Conclusions

Behavioral addiction in digital environments may reflect not only excessive engagement with specific activities, but a broader reorganization of motivational dynamics shaped by interactions between neurobiological processes and engineered reinforcement environments. In the present framework, maladaptive persistence is conceptualized as an emergent property of a coupled brain–environment system whose reward landscape may become progressively distorted under sustained high-density and high-variance reinforcement.

Reward Instability Theory extends existing accounts of addiction by shifting the unit of analysis from isolated behaviors or localized mechanisms toward system dynamics. Within this perspective, persistent behavioral patterns may arise through convergence toward attractor-like states shaped by reinforcement learning, salience processes, executive regulation, and environmental reward structure. The proposed BRII is intended as a heuristic systems construct for describing relative instability rather than as a validated diagnostic instrument.

Although primarily theoretical, the framework generates empirically testable predictions regarding non-linear change, declining behavioral variability, persistence dynamics, and early warning signals of reduced flexibility. Evaluating these predictions will require longitudinal, multimodal, and person-centered approaches capable of capturing behavior as a dynamic process unfolding over time.

More broadly, the present work suggests that the effects of digital environments on human behavior may not be fully understood without considering how platform-driven reinforcement structures shape motivational systems. Integrating neurobiological, behavioral, and environmental perspectives within a common dynamical framework may help advance future research in addiction science, digital mental health, and computational psychiatry. While developed with particular emphasis on digital environments, the underlying systems logic may also prove informative for other forms of behavioral addiction—including gambling disorder, compulsive shopping, and problematic food consumption—in which reinforcement density, salience, and reduced behavioral flexibility interact over time.

## Figures and Tables

**Table 1:** Neurobiological and individual factors influencing reward system dynamics in the Reward Instability framework.

Factor	Neurobiological Mechanism	System-Level Effects	Reward Landscape Impact
Dopaminergic signaling (genetic variation, e.g., *DRD2*, *SLC6A3*)	Modulation of reward prediction error encoding and synaptic plasticity	Modulates reinforcement learning gain and sensitivity to reward gradients	Steepens reward gradients, increasing convergence toward high-reward states
Prefrontal regulation (e.g., *COMT*)	Regulation of executive control and top-down modulation of behavior	Modulates capacity for behavioral inhibition and goal-directed control	Expands or constrains accessibility of alternative behavioral trajectories
Impulsivity and delay discounting traits	Reduced delay discounting thresholds and increased sensitivity to immediate rewards	Biases decision-making toward short-term reinforcement	Shifts system toward shallow but rapidly accessible reward peaks
Stress and allostatic load	Dysregulation of baseline reward processing and stress-related neuroadaptation	Alters baseline reward sensitivity and increases reliance on habitual responding	Globally deforms the reward landscape, reducing salience of alternative rewards
Salience attribution networks (dopaminergic–insula interactions)	Enhanced cue-triggered motivational salience	Increases attentional capture and cue-driven behavior	Amplifies prominence of specific reward peaks, reinforcing attractor formation

Examples are illustrative and not exhaustive.

**Table 2:** Candidate proxies for operationalizing the Behavioral Reward Instability Index (BRII).

BRII Dimension	Candidate Proxies	Data Sources	System Role	Expected Dynamic Effect
Individual Reward Sensitivity (IRS)	Impulsivity indices, delay discounting, neurocognitive performance	Behavioral tasks, cognitive testing apps	Modulates sensitivity to reward signals and amplification of reward gradients	Higher IRS may amplify responsiveness to reinforcement under high DRE
Digital Reward Exposure (DRE)	Screen time, notification frequency, short-form content exposure	Smartphone logs, app usage analytics	Shapes density and variability of environmental reinforcement	Higher DRE may accelerate convergence toward dominant reward states
Behavioral Variability (BV)	Behavioral entropy, activity diversity, sleep regularity	Wearables, GPS, app diversity metrics	Maintains distributed engagement and counteracts attractor formation	Lower BV may reduce resilience and favor convergence
Temporal Dynamics (BRII(t))	Fluctuations in activity patterns, recovery from perturbation, variance shifts	Longitudinal behavioral data	Captures time-dependent evolution of instability	Early warning signals may include increased variance and critical slowing down

Examples are illustrative and not exhaustive. Proxies require empirical validation.

## Data Availability

No new data were created or analyzed in this study. Data sharing is not applicable to this article.

## Supplementary Materials

The following supporting information can be downloaded at: https://www.mdpi.com/article/10.3390/brainsci16060584/s1, Figure S1: BRII-informed clinical mapping and 3Z intervention framework. Figure S2: Distribution of BRII scores in a simulated population (N = 100). Table S1: Qualitative system classification. Table S2: Parameter-oriented intervention logic.
