# Supplementary material for: Neural Reward Processing in Digital Addiction: A Dynamical Systems Theory of Reward Instability
Source: Brain Sci. 2026 May 29;16(6):584. doi: 10.3390/brainsci16060584 (PMC13296752; doi:10.3390/brainsci16060584)
Supplement: Supplementary file 1 [file brainsci-16-00584-s001.zip › brainsci-4308346-supplementary.pdf]

Supplementary S1. BRII-Informed Clinical Mapping and 3Z Translational Pilot (Proof-of-Concept Version)

All formulations in this supplement are heuristic and illustrative, and are intended solely to support hypothesis generation rather than clinical classification or decision-making.

S1.1 Purpose

This supplementary pilot presents a proof-of-concept–oriented translational extension of Reward Instability Theory, providing a structured framework for mapping system-level reward instability into clinically interpretable assessment and intervention pathways.

The model integrates:

- the Behavioral Reward Instability Index (BRII) as a system-level descriptor, and
- the 3Z motivational architecture (Understand–Desire–Act) as a staged behavioral response aligned with system dynamics.

This framework is designed as a translational scaffold supporting early-stage behavioral modeling, hypothesis generation, and exploratory intervention alignment. It is not intended as a diagnostic or therapeutic instrument.

Importantly, this supplement extends the formal framework introduced in the main manuscript by providing a minimal operational pathway linking system dynamics to applied behavioral interpretation.

S1.2 Conceptual Integration

Within the Reward Instability framework, behavioral dysregulation is conceptualized as a dynamic transition toward attractor-dominated regimes, driven by:

- increased reinforcement density and variability (↑ DRE)
- elevated reward sensitivity (↑ IRS)
- reduced behavioral variability (↓ BV)

The BRII captures this instability as a non-linear system variable, while the 3Z architecture translates system states into phase-aligned intervention logic.

This integration allows system-level instability to be interpreted not as a static condition, but as a trajectory evolving across stability regimes.

The relationship between system-level instability and intervention phases is illustrated schematically in Figure S1.

S1.3 Translational Workflow and Operational Formalization

To support translational applicability, the BRII-informed framework can be represented as a structured workflow linking behavioral data, system-level approximation, and intervention alignment.

This workflow comprises six core stages:

1. Data Acquisition

   Passive behavioral data collected via smartphones and wearable devices

2. Proxy Extraction

   Approximation of BV, DRE, and IRS using behavioral indicators

3. Feature Normalization

   All proxy measures are scaled to a common range (e.g., 0–1) to enable cross-domain comparability

4. System Approximation

   Semi-quantitative estimation of system instability using normalized parameters

5. State Classification

Assignment to stability regime (stable / transitional / attractor-dominated)

6. Longitudinal Monitoring

Tracking of behavioral variability, repetition, and disengagement dynamics over time

Operational Representation

For translational purposes, system instability may be approximated using a normalized composite index:

$BRII(t) = (IRS_n \times DRE_n) / (BV_n + \varepsilon)$

where:

- $IRS_n \in [0,1]$ — normalized reward sensitivity
- $DRE_n \in [0,1]$ — normalized digital reward exposure
- $BV_n \in [0,1]$ — normalized behavioral variability
- ε — small positive constant (e.g., 0.01) to prevent division by zero

This formulation provides a computationally tractable approximation of system-level instability while preserving the non-linear interaction structure proposed in the main model.

Interpretation as a Control Parameter

Within a dynamical systems framework, BRII(t) may be interpreted as a control parameter governing system regime:

- Low BRII → distributed behavioral regime
- Intermediate BRII → transitional instability
- High BRII → attractor-dominated regime

Rather than defining fixed thresholds, regime boundaries may be estimated empirically based on population distributions or longitudinal change within individuals.

Dynamic Update (Temporal Form)

To capture temporal evolution, BRII may be evaluated over sliding windows:

$BRII(t+\Delta t) = f(IRS_n(t), DRE_n(t), BV_n(t))$

where Δt represents a defined observation window (e.g., daily or weekly aggregation).

This formulation allows detection of:

- increasing instability trajectories
- reduced recovery after perturbation
- early signs of convergence toward dominant states

Minimal Scoring Implementation (PoC Layer)

For proof-of-concept applications, proxy variables may be approximated as:

- $DRE_n \approx$ normalized(screen time + notification frequency + session fragmentation)
- $BV_n \approx$ normalized(activity entropy + behavioral diversity + sleep variability)
- $IRS_n \approx$ normalized(impulsivity + delay discounting + response to reward cues)

This allows BRII to be computed as a composite index from passive and behavioral data streams, enabling exploratory modeling in real-world environments.

S1.4 Rapid Clinical Mapping (Heuristic Assessment)

A brief structured assessment may be used to approximate system state.

Behavioral Variability (BV)

Indicators:

- reduced diversity of daily activities
- repetitive behavioral patterns
- difficulty disengaging

Interpretation:

- high BV → distributed reward engagement (stable regime)
- low BV → reduced flexibility and increased attractor risk

Digital Reward Exposure (DRE)

Indicators:

- frequent checking behavior
- high notification frequency
- repeated short engagement cycles

Interpretation:

- high DRE → strong environmental reinforcement pressure
- increased convergence within reward landscape

Reward Sensitivity (IRS, proxy)

Indicators:

- impulsivity
- strong response to immediate reward
- difficulty delaying gratification

Interpretation:

- high IRS → amplification of reinforcement effects
- increased susceptibility to rapid convergence

S1.5 Table S1. Qualitative System Classification

System State	Behavioral Characteristics
Stable	High variability, distributed engagement
Transitional	Reduced variability, increasing repetition
Attractor-dominated	Low variability, persistent behavioral loops, reduced disengagement

This classification is conceptual and non-diagnostic.

S1.6 BRII-to-3Z Translational Mapping

The 3Z architecture operationalizes system instability into three functionally distinct intervention domains:

Understand

Phase: Emerging instability

Objective: Increase awareness of behavioral patterns and reinforcement structure

Mechanism: Makes implicit reward dynamics cognitively accessible

Desire

Phase: Attractor formation

Objective: Shift motivational salience toward alternative value structures

Mechanism: Reweights the reward landscape

Act

Phase: Attractor stabilization

Objective: Restore behavioral variability and disrupt repetitive loops

Mechanism: Redistributes reinforcement across behavioral domains

S1.7 Table S2. Parameter-Oriented Intervention Logic

Parameter	System Dysfunction	Intervention Focus
DRE	Excessive reinforcement density	Reduce exposure to high-frequency digital stimuli
BV	Behavioral rigidity	Increase behavioral diversity
IRS	Elevated reward reactivity	Strengthen regulatory control

S1.8 Temporal Monitoring and Early Warning Signals

The framework emphasizes longitudinal tracking of system dynamics, including:

- progressive reduction in behavioral variability
- increasing behavioral repetition
- reduced disengagement capacity
- slower recovery following behavioral perturbation

These patterns may indicate proximity to critical transitions, consistent with early warning signals observed in complex systems.

S1.9 Illustrative Use Case (Proof-of-Concept Scenario)

An individual exhibits:

- high notification frequency and fragmented engagement cycles (↑ DRE)
- reduced behavioral diversity (↓ BV)
- elevated impulsivity (↑ IRS)

System interpretation: transitional → attractor-prone regime

Intervention alignment:

- *Understand*: awareness of reinforcement loops
- *Desire*: revaluation of alternative rewards
- *Act*: structured diversification of behavior

Observed changes (hypothetical):

- increase in BV
- reduction in DRE
- improved disengagement capacity

S1.10 Scope and Limitations

This pilot represents a conceptual and exploratory proof-of-concept framework and is not validated for clinical use.

Behavioral indicators are indirect proxies requiring:

- empirical calibration
- validation across populations
- longitudinal testing

S1.11 Synthesis

The integration of BRII and the 3Z architecture provides a bridge between system-level theory and structured behavioral interpretation, enabling instability to be conceptualized as a dynamic process and interventions to be aligned with system parameters.

Figure S1. BRII-informed clinical mapping and 3Z intervention framework.

The figure illustrates the relationship between system-level reward instability and phase-specific intervention. Core parameters—behavioral variability (BV), digital reward exposure (DRE), and reward sensitivity (IRS)—determine system state along a continuum from stable to attractor-dominated regimes. The 3Z architecture provides a structured intervention pathway aligned with system dynamics. Behavioral change is conceptualized as modulation of reward landscape topology rather than direct suppression of behavior.

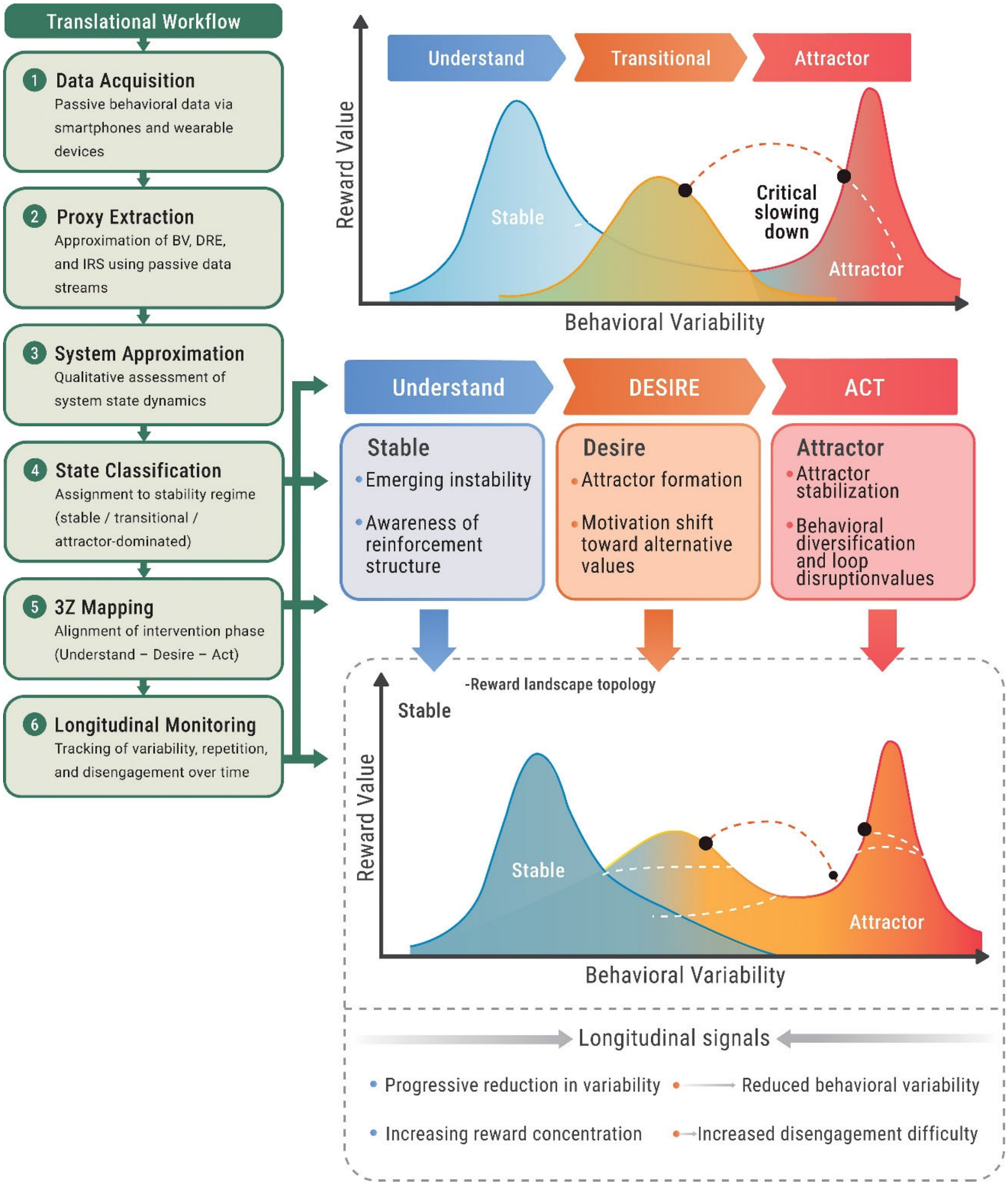

S1.12 Simulated Population-Level Application of BRII

To illustrate how the BRII framework can be used to identify population-level trends in problematic digital engagement, we present a simulated dataset of 100 hypothetical digital users with varying behavioral profiles.

Simulation Parameters

Each simulated user was assigned values for the three core BRII components, drawn from realistic distributions:

- Reward Sensitivity (IRS): Sampled from a normal distribution, $\mu = 0.5$, $\sigma = 0.15$, truncated to
- Digital Reward Exposure (DRE): Sampled from a beta distribution, $\alpha = 2$, $\beta = 3$, reflecting right-skewed exposure patterns typical of digital platform use
- Behavioral Variability (BV): Sampled from a beta distribution, $\alpha = 3$, $\beta = 2$, reflecting typical population variability in behavioral repertoires

BRII scores were computed for each individual using the formula: BRII = (IRS × DRE) / (BV + 0.01)

Results

Figure S2 presents the distribution of BRII scores across the simulated population. The distribution shows:

- Low/adaptive cluster (BRII < 0.3, n = 55): Individuals with distributed behavioral patterns, low digital reinforcement exposure, or high behavioral diversity—consistent with adaptive digital engagement
- Moderate/transitional cluster (0.3 ≤ BRII < 0.7, n = 36): Transitional profiles with intermediate vulnerability
- High/vulnerable cluster (BRII ≥ 0.7, n = 9): Individuals combining high reward sensitivity, high digital exposure, and low behavioral diversity—a profile consistent with problematic engagement patterns observed in empirical addiction research

Interpretation

This simulation demonstrates that the BRII framework can identify population subgroups at elevated risk for problematic digital engagement based on the interaction of individual neurobiological traits (IRS), environmental reinforcement structure (DRE), and behavioral flexibility (BV).

Importantly, BRII is not a diagnostic tool for individual classification. Rather, it serves as a population-level research instrument for:

- Identifying vulnerability trends across groups
- Tracking longitudinal shifts in system-level risk
- Guiding targeted prevention strategies toward high-risk subpopulations
- Generating hypotheses for empirical validation studies

Limitations

This simulation uses arbitrary but plausible parameter distributions. Real-world applications would require:

- Empirical calibration of proxy measures
- Validation against clinical and behavioral outcomes
- Longitudinal data to assess temporal stability
- Cross-cultural validation to ensure generalizability

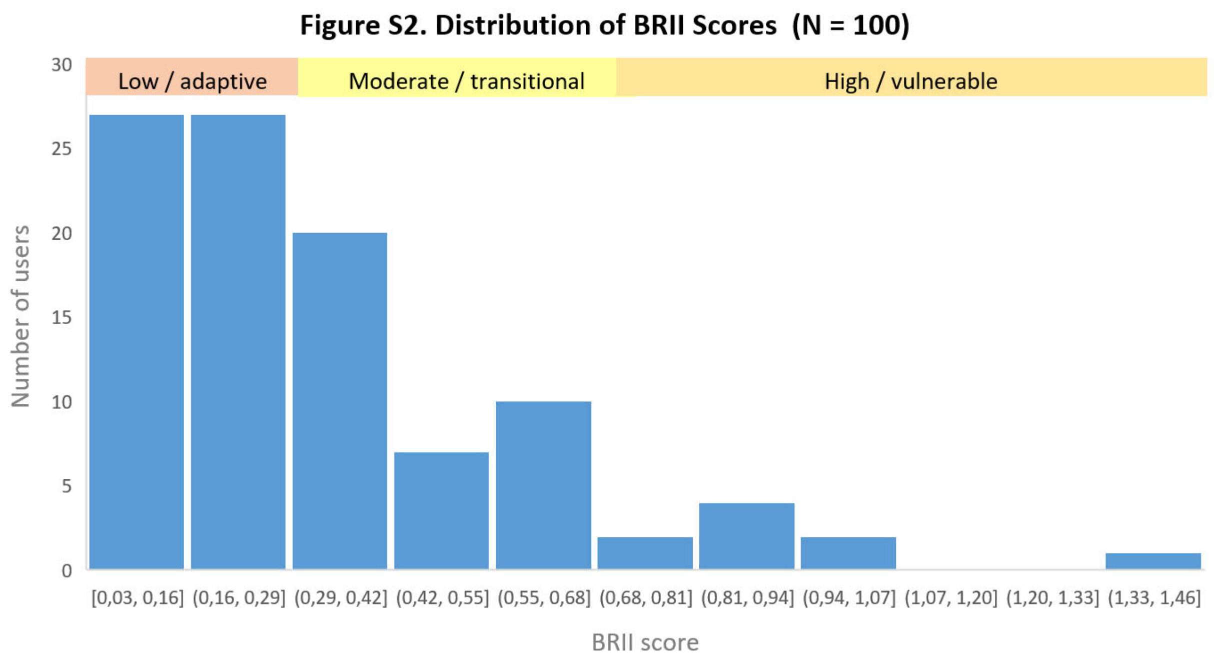

Figure S2. Distribution of BRII scores in a simulated population (N = 100). Histogram bars show frequency distribution; background shading indicates three heuristic risk zones: low/adaptive (n = 55), moderate/transitional (n = 36), and high/vulnerable (n = 9). Zone boundaries are at BRII = 0.3 and 0.7. This proof-of-concept simulation illustrates how the BRII framework may identify population-level vulnerability gradients; it is not intended for individual diagnostic classification.
